# Clinical evaluation of chitosan/polycaprolactone nanofibrous scaffolds releasing tetracycline hydrochloride in periodontal pockets of patients with chronic periodontitis

**DOI:** 10.34172/japid.2023.023

**Published:** 2023-12-03

**Authors:** Janet Moradi Haghgoo, Parviz Torkzaban, Parisa Hashemi, Rana Sarvari, Sana Hashemi, Elahe Fakhri, Behnaz Alafchi

**Affiliations:** ^1^Department of Periodontics, School of Dentistry, Hamadan University of Medical Sciences, Hamadan, Iran; ^2^Infectious and Tropical Diseases Research Center, Tabriz University of Medical Sciences, Tabriz, Iran; ^3^Department of Prosthodontics, School of Dentistry, Tabriz University of Medical Sciences, Tabriz, Iran; ^4^Dental and Periodontal Research Center, Faculty of Dentistry, Tabriz University of Medical Sciences, Tabriz, Iran; ^5^Department of Biostatistics, School of Public Health, Hamadan University of Medical Sciences, Hamadan, Iran

**Keywords:** Local drug delivery, Nanofibrous films, Periodontal pocket, Periodontitis

## Abstract

**Background.:**

The role of bacteria in the initiation and progression of periodontitis has led to a great interest in using antibiotics to suppress pathogenic microbiota. Considering the drawbacks of systemic antibiotics’ application, local delivery systems directly in the periodontal pocket can be helpful. Therefore, the effect of an efficient tetracycline-loaded delivery system was investigated on the clinical parameters of periodontitis.

**Methods.:**

In this clinical trial with a split-mouth design, 10 patients with periodontitis with pocket depths≥5 mm were included. After scaling and root planing (SRP) for all the patients, one side of the mouth was randomly considered as the control group, and on the other side, chitosan/polycaprolactone (PCL) nanofibrous films containing tetracycline (5%) were placed in pockets of 5 mm and deeper. Clinical measurements of pocket probing depth (PPD), clinical attachment loss (CAL), and bleeding on probing (BOP) indices were made at the beginning and after 8 weeks of intervention. PPD, CAL, and BOP parameters were compared between the control and test groups before and after the intervention with paired *t* tests using SPSS 24. The significance level of the tests was considered at *P*<0.05.

**Results.:**

The mean PPD, CAL, and BOP in both the control (SRP) and test (LDDs) groups decreased after 8 weeks. A significant difference was detected in reducing PPD, BOP, and CAL after 8 weeks in 5-mm pockets, and the mean values were higher in the test group than in the control (*P*<0.05).

**Conclusion.:**

The local drug delivery system using chitosan/PCL nanofibrous films containing tetracycline can effectively control periodontal diseases by reducing pocket depth and inflammation and improving CAL without offering side effects, although further evaluations are needed.

## Introduction

 Periodontitis is a multifactorial inflammatory disease leading to the destruction of periodontal ligament tissue, alveolar bone, and tooth loss. The primary cause of periodontal disease is dental plaque, containing colonies of *Porphyromonas gingivalis* and *Aggregatibacter actinomycetemcomitans*.^1,2^ Dental plaque results in inflammation in surrounding tissues since the bacteria can produce enzymes that destroy the supporting connective tissue.^3^

 The common non-surgical therapeutic approach includes mechanical scaling and root planing (SRP). The bacterial nature of periodontitis indicates the reason for the widespread prescription of antibiotics as an adjunct to SRP. When administering systemic antibiotics, since not all antibiotics can reach the periodontal pocket in a therapeutic dose, mainly due to insufficient tissue penetration, the final target concentration is not reached.^4^ Contrary to systematic antibiotic therapy, controlled drug release systems are advantageous due to reduced side effects, toxicity, and high efficiency. In recent years, attention has been drawn to drug delivery technology tremendously. In this regard, nanofibrous scaffolds fabricated by electrospinning have been widely used as promising delivery carriers of antibiotics, limiting bacterial adhesion and biofilm formation and promoting bone and tissue regeneration.^5,6^

 Polycaprolactone (PCL) is a Food and Drug Administration (FDA)-approved synthetic polymer and is considered one of the most applicable polymers used for the development of drug delivery systems.^7,8^ Chitosan, on the other hand, is a natural linear cationic polysaccharide composed of randomly distributed β-(1→4)-linked D-glucosamine (deacetylated unit) and *N*-acetyl-D-glucosamine (acetylated unit) with properties such as high biocompatibility, moisture absorption, mucoadhesiveness and biodegradability.^9^

 Tetracycline provides broad-spectrum antimicrobial activity and is considered the most common antimicrobial drug used in periodontal therapy.^10^ Previous studies suggest that in addition to the antimicrobial effect, tetracycline inhibits tissue collagenase, delaying the degradation of collagen, as seen in periodontal disease.^10,11^

 Thus, this study aimed to incorporate tetracycline hydrochloride into nanofibers of PCL and chitosan to provide a more sustained and prolonged release of the drug and investigate the efficiency of the fabricated carrier on the clinical parameters of patients with periodontitis.

## Methods

 This randomized controlled clinical trial compared the clinical parameters in subjects who underwent non-surgical periodontal therapy along with a local application of chitosan/PCL nanofibers containing 5% tetracycline (prepared as previously described^12^) and subjects who undergone non-surgical periodontal therapy alone. The design of this study was approved by the Ethics Committee of Hamadan University of Medical Sciences (approval code: IR.UMSHA.REC.1401.466).

 The present study included 10 patients referred to the Periodontics Department of Hamedan University of Medical Sciences. Patients diagnosed with chronic periodontitis without any systemic health problems who had not received any surgical or non-surgical periodontal therapy in the past 6 months were included. Individuals with a history of using mouthwashes 3 months before the study or patients with systemic diseases, alcohol and cigarette use, or allergy to tetracycline or lidocaine were excluded from the study. The patients signed informed consent forms; then, each patient received hygiene instructions emphasizing the Bass brushing technique.

 The clinical parameters recorded were the pocket probing depth (PPD) using a Williams periodontal probe (Hu-Friedy, USA), bleeding on probing (BOP), and clinical attachment loss (CAL). After recording clinical parameters from each side of all the posterior teeth (mesiobuccal, midbuccal, distobuccal, mesiolingual, midlingual, and distolingual) except for the third molar at baseline, a thorough SRP with a piezoelectric ultrasonic scaler (Woodpecker UDS-K, China) was performed in both groups. The clinical parameters were assessed at baseline and 8 weeks after receiving the treatment.

 In each patient, the mouth was divided into two segments, which were randomly assigned to two groups:

1. Control group: 10 sites treated with SRP alone
2. Test group: 10 sites treated by SRP and tetracycline containing nanofibers in pockets with a probing depth of ≥ 5 mm

**Figure 1:**
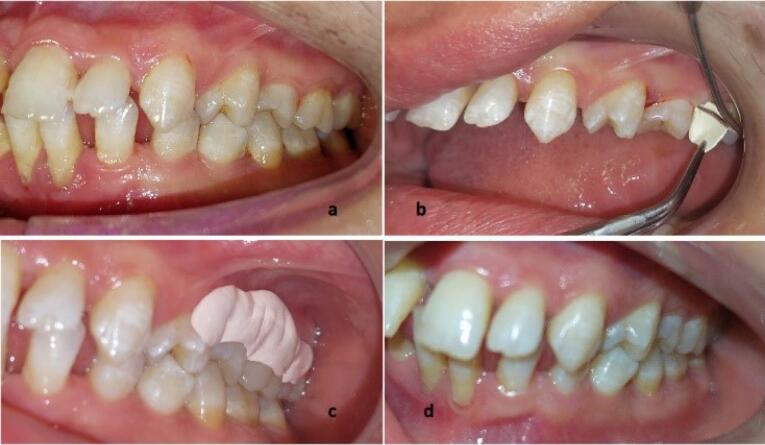
The clinical view of oral condition before SRP (a), while placing the scaffold (b), after placing the Coe-Pak (c), and after 8 weeks (d)

Fibers were gently placed in pockets using a Williams probe. No excess pressure was applied while placing the materials to avoid any trauma. After adapting to the gingival tissue, the gingival margin was sealed with Coe-Pak for a week to prevent the dislodgement of the drug and the ingress of oral fluids (Figure 1).^13^

## Results

 According to the independent sample t-test, there were no significant differences in the mean PPD, CAL, and BOP between the control and experimental groups in the maxilla and mandible before (Table 1) and 8 (Table 2) weeks after the intervention (*P* > 0.05).

**Table 1:** Mean PPD, BOP, and CAL in the control and experimental groups at baseline

		**Mean**	**Standard Deviation**	**t-value**	***P***** value**
PPD up	Control	4.04	0.62	0.18	0.85
	Treatment	3.99	0.45		
PPD down	Control	3.56	0.35	-0.26	0.79
	Treatment	3.60	0.25		
PPD Total	Control	3.80	0.45	0.24	0.98
	Treatment	3.80	0.30		
CAL up	Control	2.65	0.97	0.21	0.83
	Treatment	2.54	0.74		
CAL down	Control	2.18	0.73	-0.15	0.87
	Treatment	2.23	0.55		
CAL	Control	2.41	0.81	0.05	0.95
	Treatment	2.39	0.61		
BOP up	Control	70.41	19.08	-0.14	0.88
	Treatment	71.66	19.02		
BOP down	Control	75.41	13.09	-0.07	0.93
	Treatment	75.83	10.35		
BOP	Control	72.91	11.82	-0.17	0.86
	Treatment	73.75	9.73		

PPD, pocket probing depth; CAL, clinical attachment loss; BOP, bleeding on probing.

**Table 2:** Mean PPD, BOP, and CAL in the control and experimental groups after 8 weeks

		**Mean**	**Standard Deviation**	**t-value**	***P***** value**
PPD up	Control	3.38	0.56	1.73	0.85
	Treatment	3.03	0.32		
PPD down	Control	2.95	0.34	0.75	0.79
	Treatment	2.85	0.27		
PPD	Control	3.17	0.41	1.49	0.98
	Treatment	2.94	0.25		
CAL up	Control	2.03	0.89	0.89	0.83
	Treatment	1.72	0.65		
CAL down	Control	1.64	0.66	0.40	0.87
	Treatment	1.53	0.56		
CAL	Control	1.84	0.75	0.70	0.95
	Treatment	1.62	0.60		
BOP up	Control	40.41	16.90	2.97	0.88
	Treatment	20.41	12.94		
BOP down	Control	33.75	6.03	6.20	0.93
	Treatment	15.00	7.40		
BOP	Control	37.08	8.99	5.29	0.86
	Treatment	17.70	7.29		

PPD, pocket probing depth; CAL, clinical attachment loss; BOP, bleeding on probing.

 According to the paired-sample t-test, in both the control and experimental groups, there were significant differences in the mean PPD, CAL, and BOP after the intervention between the maxilla and mandible (*P* < 0.001).

 According to the paired-sample t-test, in both the control and experimental groups, there were significant differences in the mean PPD, CAL, and BOP before (Table 3) and after (Table 4) the intervention in all the angles of pockets ≥ 5 mm (*P* < 0.001).

**Table 3:** Mean PPD and CAL in pockets ≥ 5 mm in the control and experimental groups at baseline

		**Mean**	**Standard Deviation**	**t-value**	***P***** value**
PPD Mesiobuccal	Control	5.75	0.91	0.51	0.61
	Treatment	5.64	0.87		
PPD Distobuccal	Control	5.59	1.03	0.97	0.33
	Treatment	5.40	0.74		
PPD Mesiolingual	Control	5.14	0.45	0.00	1.00
	Treatment	5.14	0.36		
PPD Distolingual	Control	5.29	0.54	1.13	0.26
	Treatment	5.15	0.36		
CAL Mesiobuccal	Control	4.62	1.12	1.65	0.10
	Treatment	4.21	1.00		
CAL Distobuccal	Control	4.23	1.16	0.47	0.63
	Treatment	4.12	0.99		
CAL Mesiolingual	Control	3.96	0.70	1.00	0.31
	Treatment	3.77	0.64		
CAL Distolingual	Control	3.92	0.91	1.05	0.29
	Treatment	3.71	0.58		
PPD before	Control	5.51	0.66	0.69	0.49
	Treatment	5.42	0.54		
CAL before	Control	4.32	0.87	1.31	0.19
	Treatment	4.08	0.73		

PPD, pocket probing depth; CAL, clinical attachment loss.

**Table 4:** Mean PPD and CAL in pockets ≥ 5 mm in the control and experimental groups after 8 weeks

		**Mean**	**Standard Deviation**	**t**	***P***** value**
PPD Mesiobuccal	Control	4.93	0.66	5.89	< 0.001
	Treatment	4.04	0.62		
PPD Distobuccal	Control	4.73	0.79	4.97	< 0.001
	Treatment	3.97	0.57		
PPD Mesiolingual	Control	4.44	0.75	3.31	< 0.001
	Treatment	3.81	0.40		
PPD Distolingual	Control	4.44	0.64	4.39	< 0.001
	Treatment	3.78	0.49		
CAL Mesiobuccal	Control	4.03	1.03	5.07	< 0.001
	Treatment	2.83	0.98		
CAL Distobuccal	Control	3.40	1.01	2.62	< 0.001
	Treatment	2.82	0.98		
CAL Mesiolingual	Control	3.29	0.86	2.79	< 0.001
	Treatment	2.66	0.78		
CAL Distolingual	Control	3.18	0.92	3.11	< 0.001
	Treatment	2.46	0.84		
PPD after	Control	4.66	0.52	6.93	< 0.001
	Treatment	3.97	0.37		
CAL after	Control	3.55	0.79	4.36	< 0.001
	Treatment	2.82	0.74		

PPD, pocket probing depth; CAL, clinical attachment loss.

 The reduced pocket depth, CAL gain, and the mean difference in the BOP values were significantly higher after placing the tetracycline-containing fibers (1.44, 1.26, and 51.78, respectively) compared to the control group (0.85, 0.76, and 13.09, respectively) (*P* < 0.05).

 According to the independent samples *t* test, there were no significant differences in the mean PPD, CAL, and BOP in all angles of pockets ≥ 5 mm between the control and experimental groups before the intervention (*P* > 0.05) (Tables 3 and 5). However, there were significant differences between the mentioned parameters between the control and experimental groups after 8 weeks (*P* < 0.001) (Tables 4 and 5).

**Table 5:** Mean BOP in pockets ≥ 5 mm in the control and experimental groups at baseline and after 8 weeks

		**Mean**	**Standard Deviation**	**t-value**	***P***** value**
BOP before per.	Control	76.19	33.10	0.27	0.78
	Treatment	74.40	26.76		
BOP after per.	Control	63.09	33.69	6.29	< 0.001
	Treatment	22.06	24.57		

BOP, bleeding on probing.

## Discussion

 Periodontal diseases are among the most common and well-known dental diseases affecting patients’ lives by disrupting comfort and function. Therefore, the treatment of periodontal problems is of great importance. Bacterial accumulation and plaque formation are the paramount causes of gingivitis and periodontitis; therefore, plaque control seems to be an essential step in the treatment plan. Conventional non-surgical treatments, including mechanical methods of plaque and calculus removal, are insufficient and do not lead to the satisfactory reconstruction of soft and hard tissues. Furthermore, local antimicrobial therapy has been proposed as an adjunctive treatment for mechanical plaque removal.^5^ Previous studies have introduced various delivery systems such as hydrogels and sponges; however, an ideal carrier has not been proposed yet.^9,14^ In this regard, electrospinning has been introduced as a promising technique for developing effective carriers for treating various oral diseases, including ulcers, infections, and periodontitis. Nanofibers act as potential drug reservoirs, provide a good environment for cellular adhesion, and promote bone and tissue regeneration.^15,16^ Moreover, considering the conditions in the periodontal pocket, it has been shown that electrospun nanofibrous membranes can improve adhesion to the gingiva and release the drug in direct contact with surrounding tissues, prolonging the therapeutic effect.^17^ Therefore, in the present study, a nanofibrous chitosan/PCL scaffold was developed to deliver tetracycline into the periodontal pocket for local antibacterial therapy, and the clinical evaluation was carried out.

 The nanofibrous scaffolds were placed in the periodontal pockets after SRP at the baseline, and patients were recalled for follow-up after 8 weeks. Patients had no complaints regarding the comfort of the placed material, and no irritation or inflammation at the site was observed. The results of the present study suggest that applying tetracycline-containing nanofibers combined with SRP is beneficial in treating chronic periodontitis and improves periodontal parameters (CAL, PD, and BOP) for 8 weeks. It was shown that local antibiotic therapy as an adjunct to conventional SRP treatment decreases the CAL, BOP, and periodontal pocket depth in pockets ≥ 5 mm significantly higher than SRP alone (*P* < 0.001). Although SRP decreased the evaluated clinical parameters, local antibiotic therapy demonstrated more efficient results (*P* < 0.001).

 Improvements in therapeutic efficacy due to the combined effect of both local antibiotic therapy using a nanofibrous delivery device and mechanical removal of the periodontal pathogens have been previously reported in similar studies. According to a study by Khan et al,^13^ placing chitosan films containing metronidazole and levofloxacin in periodontal pockets ( ≥ 5 mm) following SRP treatment reduced PPD, GI, BOP, and CAL. Although chitosan films are biocompatible and act as an acceptable reservoir for antibiotics, their presence in periodontal pockets is short due to high biodegradability.^9^ Application of chitosan in this study was to increase the biodegradability, biocompatibility, and mucoadhesiveness of developed films.^14^ Previous studies showed that chitosan in the forms of gels,^18^ films,^13^ and sponges^19^ can be used to reduce the periodontal depth and control BOP. Khajuria et al^20^ developed a metformin-loaded chitosan film using the solvent-casting technique and showed that chitosan was an acceptable carrier to deliver metformin to deep periodontal pockets for 11 days. As mentioned above, nanofibrous carriers act as a more advantageous matrix in periodontal pockets. Previous clinical studies have demonstrated the efficacy of fibers as drug-delivery systems in treating periodontal diseases.^12,21,22^ These studies reported significant improvements following treatment with a combination of mechanical plaque and calculus removal and fiber insertion during a long-term period and have demonstrated the clinical efficacy of fibers in reducing attachment loss and, consequently, the probing depth.^21^ Notwithstanding, previous systematic reviews emphasized a lack of evidence to support established clinical protocols.^23^ Furthermore, some studies showed that local antibiotic therapy might provide short-term benefits in controlling inflammation, but in the long term, it caused no difference.^24,25^

 Antibiotic-loaded PCL has been introduced as a promising drug delivery system for the treatment of periodontitis.^26^ In this study, a synthetic polymer such as PCL was added to chitosan as previously described^12^ to decrease the biodegradability and increase the existence of the film in the periodontal pocket, prolonging the therapeutic effect. In a preliminary clinical study, Khan et al^12^ demonstrated the therapeutic potential of chitosan/PCL nanofibrous membranes containing tinidazole compared to SRP in patients with periodontitis. Consistent with our study, tinidazole-loaded chitosan/PCL placement in pockets > 5 mm in depth following SRP reduced the PD and improved the CAL in 8 weeks. Furthermore, Khan et al^12^ showed that the developed carrier significantly reduced the BOP than SRP alone. In the present study, tetracycline was chosen to be released in the pockets due to its anti-collagenase activity and antibacterial efficacy.^10^

 From this clinical study, it could be concluded that the chitosan/PCL nanofibrous membranes showed no signs of irritation at the treatment site. The traditional mechanical technique, including SRP, effectively improves clinical parameters in patients with periodontitis. Patients treated with the medicated nanofibrous membrane in addition to SRP demonstrated significant improvement compared to the patients treated with SRP alone. Notwithstanding, the limitations of this study, including a small sample size and limited follow-up duration, suggest further investigations to bring the fabricated nanofibrous membranes to widespread clinical applications.

## Conclusion

 This clinical trial on patients with periodontitis demonstrated the therapeutic efficacy of the tetracycline-loaded chitosan/PCL nanofibrous membrane. Applying chitosan/PCL nanofibrous membrane following SRP reduced the PPD, CAL, and BOP by the slow release of tetracycline; however, further trials and investigations are necessary considering the limitations of this study.

## Competing Interests

 The authors declare that they have no competing interests.

## Data Availability Statement

 The datasets used and/or analyzed during the current study are available from the corresponding author upon reasonable request.

## Ethical Approval

 This study was approved by the Research Ethics Committee of Hamedan University of Medical Sciences with the reference number IR.UMSHA.REC.1401.466.

## Funding

 This study was supported by the Hamedan University of Medical Studies.
